# Using Nanochelating Technology for Biofortification and Yield Increase in Rice

**DOI:** 10.1038/s41598-020-60189-x

**Published:** 2020-03-09

**Authors:** Saideh Fakharzadeh, Maryam Hafizi, Mohammad Ali Baghaei, Maral Etesami, Maryam Khayamzadeh, Somayeh Kalanaky, Mohammad Esmaeil Akbari, Mohammad Hassan Nazaran

**Affiliations:** 1 Department of Research and Development, Sodour Ahrar Shargh Company, Tehran, Iran; 2 Cancer Research Centre, Shahid Beheshti University of Medical Sciences, Tehran, Iran; 3 Department of Agronomy, Faculty of Agriculture, Tarbiat Modares University, Tehran, Iran

**Keywords:** Metalloproteins, Environmental monitoring

## Abstract

Iron is a vital microelement that plays an important role in plant metabolism. Consuming a large amount of chemical fertilizers increases the risk factors of neoplastic diseases such as heavy metals and harmful components in crops edible parts. Therefore, utilizing novel technologies to increase yields without requiring more chemical fertilizers seems essential. In this regard, nanotechnology holds considerable potentials for creating valuable outputs in agriculture. The effect of nano chelated iron fertilizer, which is synthesized based on novel nanochelating technology, on agronomic traits and yield of rice were evaluated in the present study. A randomized complete block experiment was conducted with 3 replicates. The treatments were: T0 (control), T1 (2.5 g/L foliar application twice at nursery with a one-week interval), T2 (foliar application at tillering + T1), T3 (foliar application at booting + T1), T4 (foliar application at tillering and booting + T1), T5 (8 kg/ha soil application at tillering + T1), T6 (8 kg/ha soil application at booting + T1), T7 (4 kg/ha soil application at tillering + 4 kg/ha soil application at booting + T1). Nano chelated iron fertilizer increased biological yield by 27% and decreased hollow grain number by 254%; in addition, it raised protein content by 13%. This fertilizer also led to increase in nitrogen, phosphorus, potassium, iron and zinc concentrations in white rice by 46%, 43%, 41%, 25% and 50%, respectively. Nanochelating technology can decrease the need for chemical fertilizers; additionally, this technology has the capability to bio-fortify crops with vital micronutrients.

## Introduction

The climactic changes adversely impact crops and food production worldwide. Moreover, world population is continuing to rise at a rate unequal to that of food and agricultural production. The World Food Programme (WFP 2016) reports that, on average, production of crop yield per hectare is increasing slower than that of global population, and this will most likely lead to food crisis^1,2^. In addition, greenhouse effect decreases the quality of crops as it negatively affects various factors such as protein or nutrient content^3^. There is therefore a serious need for a solution that can simultaneously increase quality and quantity of crops and supply food for all humans worldwide.

In view of this, rice, as an essential strategic plant^4^, is considered the staple food for almost two-thirds of the world’s population^5^, but researchers have predicted that global warming will make paddy fields less productive in the near future. From qualitative perspective, rice is a poor source of iron, zinc and other micronutrients^6^. Although many methods, including conventional breeding, genetic engineering and agronomic approaches, have been exercised in the hope of increasing micronutrients content in rice grains^6,7^, overproduction of a variety of genetically modified crops has led to serious bio-safety challenges such as food, health and environmental safety as well as the socio-economical and ethical debates of such approaches^8^. On the other hand, crops fertilization does not necessarily increase minerals concentration in fruit, seed or grain to the desired levels^9,10^.

According to the Food and Agricultural Organization of the United Nations (FAO) report^11^, the total consumption amounts of chemical fertilizers (NPKs: nitrogen, phosphorus and potassium sources as essential fertilizers in agriculture) were 183 200 000 and 186 900 000 tons in 2013 and in 2014, respectively. These amounts reached 200 500 000 tons by the end of 2018 with a successive growth rate of 1.8 percent per year.

A long-term use of chemical fertilizers will probably cause the accumulation of toxic by-components in soil, which results in deterioration of soil ecological environment by heavy metals, nitrate and other harmful components in crops^12^. Therefore, the consumption of these contaminated products can increase the risk of various diseases, especially cancers^13–15^. As a result of this, there is an urgent need to find a safe and efficient way to increase the quality and quantity of agricultural products without any need for annual increase in chemical fertilizers consumption.

Iron is a micronutrient which has a vital role in improving crops in terms of quality and quantity, and several studies have already shown that iron fertilization^16^ has a significant influence on both parameters of rice. Nowadays, various kinds of iron compounds such as iron sulfate and iron oxide are commonly used around the world. Among these compounds, iron chelates have shown higher absorption rate^17,18^. Fe-EDDHA and Fe-EDTA are the most common forms of iron chelates; however, they have some limitations in terms of application and also absorption rate^19^. Therefore, adopting a new approach to synthesize more efficient iron chelates seems essential for delivery of iron to plants.

Nanotechnology with considerable potentials in different disciplines has been recently used in genetic enhancement, soil texture, pest and pathogens management, nutrient delivery and environmental conservation, all of which affect plant growth and development cycle^20^.

Nanochelating technology is a novel approach applied in synthesizing efficient nano structures in various disciplines. In the previous studies, the efficiency of nano chelated fertilizers that were synthesized based on this technology was proved. In this regard, Zareabyaneh *et al*. indicated that nanochelating-based nitrogen fertilizer is more resistant to leaching, has higher nitrogen use efficiency and induces more yield when compared to urea^21^. Also in another study by Ranjbar *et al*., it was revealed that applying nano calcium fertilizer designed based on the above-mentioned technology improved storage time and different quality-related parameters of apple fruit^22^. In several studies, the positive impact of applying nano chelated iron fertilizer on crop quality and quantity has already been demonstrated^23–25^.

To use the advantages of nanotechnology and reduce the application of chemical fertilizers, nano chelated iron fertilizer was used at different growth stages to evaluate the response of rice yield, some agronomic traits, grain protein content and nutrients concentration to nano chelated iron fertilizer application.

## Material and Method

### Synthesis and characterization of nano chelated iron fertilizer

Nano chelated iron fertilizer, which is synthesized based on nanochelating technology, was used in the present experiment^26^. This fertilizer was synthesized based on a method wildly known as “self-assembly”, which is thoroughly explained in a patent registered in the United States Patent and Trademark Office (USPTO)^26^. According to this method, 2.8 g of iron compound and a few drops of chloric acid are first dissolved in distilled water, and the solution is then put on the shaker to be mixed for 15 minutes. Afterwards, 3 g of organic acid and a reaction initiator are added to the solution to be mixed for further 20 minutes while the temperature of the solution does not exceed 60 °C. Following that, the solution is left for 2 hours and then is dried in a freeze dryer device before being sifted. The size of this nano structure was characterized using high-resolution transmission electron microscopy (HRTEM) images (Electron Microscopy Unit - Bio 21 device) at Molecular Science and Biotechnology Institute (The university of Melbourne, Victoria 3010, Australia).

### Study design

An experiment was conducted in the current study based on a randomized complete block design with 3 replicates at Rice Research Institute, Rasht, Iran (37.26°N, 49.58°E). To supply NPK fertilizers, 150 kg of potassium fertilizer, 150 kg of urea and 150 kg of phosphate fertilizer were applied per hectare.

Treatments were: T_0_ (control), T_1_ (foliar application twice at nursery stage with a one-week interval), T_2_ (foliar application at tillering stage + T_1_), T_3_ (foliar application at booting stage + T_1_), T_4_ (foliar application at tillering and booting stages + T_1_), T_5_ (8 kg/ha soil application at tillering stage + T_1_), T_6_ (8 kg/ha soil application at booting stage + T_1_), T_7_ (4 kg/ha soil application at tillering stage + 4 kg/ha soil application at booting stage + T_1_).

The foliar application dose of nano chelated iron fertilizer was 2.5 g per liter of water. To prepare the solution, 250 g of nano chelated iron fertilizer was dissolved in 100 liters of water and then after obtaining a homogeneous solution, foliar spraying was done in early morning.

After plowing and preparing the field, it was divided into 4 m×5 m plots which were continuously flooded with separated input and output pipes in each plot. Nitrogen fertilizer was used before planting and at tillering stage as urea source. Potassium and phosphorus fertilizers were applied as sulfate potassium and triple super phosphate sources at sowing time, respectively. After the nursery stage, the seedlings were pulled and transplanted into the main field. There was a 20 cm space between the rows with 3 plants on every hill. Thinning, weeding, irrigation and pest management were done when necessary. Finally, 12 m^2^ of each plot was harvested to record three parameters of hollow grain number, 1000-grain weight and yield. Panicle length was also measured by digital caliper at physiological maturity stage.

Nitrogen concentration was measured by Kjeldahl (1883) method^27^. Phosphorus and potassium were computed by calorimetry and flame photometry, respectively. Atomic absorption spectroscopy^28^ with a deuterium background correction by acetylene-air flame atomization was used to measure zinc, copper, iron and manganese concentrations. Moreover, an analytical line of 248.3 nm in a spectral interval of 0.2 nm was used to make the computations. Besides, the standard addition method was utilized to determine the elements concentration. Sample digestion was obtained in an MDS-2000 microwave sample-preparation system (CEM, Matthews, NC, USA) in Teflon cartridges by mixing acid (5 mL) and H_2_O_2_ (2 mL) for 20 minutes at 120 psi pressure, and eventually, the final product was evaluated directly in Teflon cartridges.

### Statistical analysis

Data were recorded and analyzed by SPSS and IRRISTAT software. The means were compared according to Duncan test at 0.01 level, and the percentage of protein was calculated by the specific coefficient (6.25) of nitrogen content.

### Ethics, consent and permissions

Rice Research Institute, Rasht, Iran (37.26°N, 49.58°E).

## Results

### Nano chelated iron fertilizer characterization

**Figure 1:**
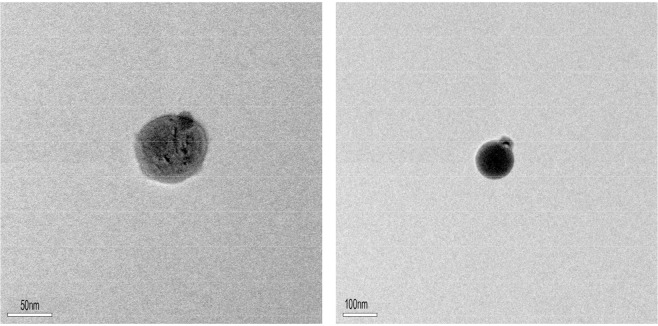
Transmission electron microscope (TEM) images.

The size of this nanocomplex was approximately 80 nm, which was determined based on TEM images by HRTEM (Fig. 1).

### Effect of nano chelated iron fertilizer on yield-related parameters and protein content

Physico-chemical characteristics and nutrients concentration of soil samples are shown in Table 1. The soil test in the present study showed that it was a silty clay soil with EC = 2.24 ds.m^−1^. The results showed (Table 2) that nano chelated iron fertilizer had significant effect (P value ≤ 0.01) on hollow grain number and 1000-grain weight, and besides paddy yield and protein content responded to the application of this fertilizer significantly (P value ≤ 0.05).

**Table 1:** Physico-chemical characteristics of soil test.

Ec Ds.m^−1^	OC (%)	N (%)	P Mg. kg^−1^	K Mg. kg^−1^	Fe Mg. kg^−1^	Mn Mg. kg^−1^	Zn Mg.kg^−1^	Cu Mg.kg^−1^	Sand (%)	Silt (%)	Clay (%)
2.24	2.74	0.210	15.1	164	21.2	14.8	7.8	6.9	11	47	42

It shows the result of the soil test conducted to determine the elements value and soil characteristics. Table 1 reveals the texture of silty loam with the level of major secondary and minor nutrients.

**Table 2:** Mean square of tiller number, plant height, panicle length, hollow grain number, 1000-grain weight, grain yield, biological yield and harvest index of rice.

Source of changes	Freedom degree	Tiller number (per plant)	Plant height(cm)	Panicle length (cm)	Hollow grain number	1000grain weight (gr)	paddy yield (kg.ha^−1^)	Biological yield (kg.ha^−1^)	Straw yield (kg.ha^−1^)	Harvest index	Protein (%)
Block	2	2.82 ns	57.72**	0.75 ns	30.43*	0.12 ns	998519.8*	2426215.8 ns	327202.8 ns	5.79 ns	0.17 ns
Fertilizers	7	2.67 ns	14.27 ns	1.87 ns	91.50**	4.51**	696184.8*	1926675.6 ns	533439.1 ns	13.98 ns	0.37*
Error	14	1.79	6.02	1.20	4.86	0.83	235044.4	1088812.7	548022.9	12.69	0.12
CV(%)		7.8	1.8	4.3	22.6	3.6	10.1	10.8	15.2	7.2	3.6

• **Significant at P ≤ 0.01, *Significant at P ≤ 0.05, ns: No significant difference.

It shows the mean square of agronomic and physiological characteristics of rice analyzed using SPSS and IRRISTAT software. According to the results, nano chelated iron fertilizer had significant effect on hollow grain number, grain weight, paddy yield and protein content.

Maximum value of tiller number (18.43 per plant), panicle length (27 cm), 1000-grain weight (28 gr), paddy yield (5845 kg/ha), biological yield (11356 kg/ha) and protein content (9.91%) were observed under T3, while minimum values of the above-mentioned parameters were obtained from control (Table 3).

**Table 3:** Mean comparison of tiller number, plant height, panicle length, hollow grain number, 1000-grain weight, paddy yield, biological yield, straw yield, harvest index and protein content of rice.

Treatments	Tiller number (per plant)	Plant height (cm)	Panicle length (cm)	Hollow grain number	1000-grain weight (gr)	paddy yield (kg.ha^−1^)	Biological yield (kg.ha^−1^)	Straw yield (kg.ha^−1^)	Harvest index (%)	Protein (%)
T0	15.53b	13.6.8b	24.5b	21.04a	23.7c	4369b	8949b	4581a	48a	8.76b
T1	17.27ab	143.1a	25.87ab	6.13d	25.7b	5023ab	9831ab	4808a	51.26a	9.79a
T2	17.8ab	137.4b	25.8ab	14.03c	25.3ab	4685b	9018b	4333a	52a	9.64a
T3	18.43a	139.7ab	27a	5.93d	28a	5845a	11356a	5511a	51.66a	9.91a
T4	17ab	137.5b	24.73b	7.5cd	26.3b	4509b	9937ab	5428a	48a	9.49a
T5	16.07ab	136.3b	25.4ab	10.73cd	25.7b	4999ab	9980ab	4981a	49.9a	9.59a
T6	16.97ab	137.9b	26ab	5.63d	25ab	4556b	9067b	4511a	50.26a	9.41a
T7	17.2ab	137.6b	25.2ab	6.73cd	25.3ab	4484b	9281b	47.97a	48.63a	9.31ab

• Treatments with the same letters do not have any significant difference.

Means were compared according to Duncan test at 0.01 level. Variation in different parameters is linked to effect of using nano chelated iron fertilizer in different stages. Results demonstrated that in most traits T3 had the maximum value.

Control and T3 had the maximum and minimum number of hollow grain, respectively (Table 3). The results also showed that nano chelated iron fertilizer increased tiller number by 18%, panicle length by 10%, 1000-grain weight by 18%, paddy yield by 30% and biological yield by 27% compared to control. Moreover, hollow grain number was decreased by 254% using this nano fertilizer.

### Effect of nano chelated iron fertilizer on rice macro and micronutrients content

**Figure 2:**
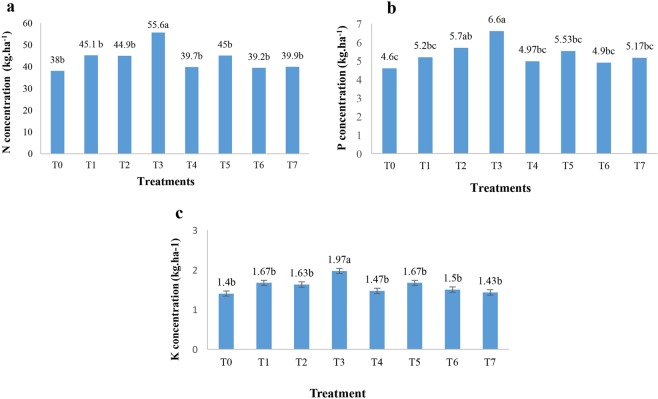
Nitrogen **(a**), phosphorus **(b)** and potassium **(c)** concentrations of white rice in response to different nano chelated iron fertilizer applications. The above-mentioned figures demonstrate the effect of nano chelated iron fertilizer on nutrients concentration which was significant on nitrogen, phosphorus and potassium concentrations of white rice.

Nano chelated iron fertilizer had significant effect on nitrogen, phosphorus and potassium concentrations of white rice as compared to control (Table 4). Figure 2a shows the effect of nano chelated iron fertilizer on nitrogen content of rice. The results showed that T3 had the maximum nitrogen concentration compared to control, while no significant difference was observed in the other treatments.

**Table 4:** Analysis of variance of N, P, K, Zn, Cu, Fe and Mn concentrations of white rice.

Source of changes	Freedom degree	N concentration (kg.ha^−1^)	P concentration (kg.ha^−1^)	K concentration (kg.ha^−1^)	Zn concentration (kg.ha^−1^)	Cu concentration (kg.ha^−1^)	Fe concentration (kg.ha^−1^)	Mn concentration (kg.ha^−1^)
Block	2	134.31**	1.81*	0.12*	0.00201**	0.00028 ns	0.00057 ns	0.00031 ns
Fertilizers	7	97.36**	1.15*	0.10**	0.00023 ns	0.00014 ns	0.00034 ns	0.00019 ns
Error	14	16.95	0.36	0.02	0.00009	0.000097	0.00045	0.0001
CV(%)		9.47	11.33	9.85	12.4	55	38.5	16.8

• **Significant at P ≤ 0.01, *Significant at P ≤ 0.05, ns: No significant difference.

Nutrients concentration of white rice was evaluated after nano chelated iron fertilizer application. Nutrients concentration analysis of white rice demonstrated that nitrogen, phosphorus and potassium responded to nano chelated iron fertilizer significantly.

Furthermore, phosphorus concentration was affected by nano fertilizers, where maximum and minimum phosphorus contents were observed in T3 (6.6 kg/ha) and control (4.6 kg/ha), respectively (Fig. 2b). Potassium concentration differed under different fertilizer treatments. According to the results (Fig. 2c), maximum potassium concentration was gained from T3, whereas control had the least potassium content. Numerically, applying nano chelated iron fertilizer increased phosphorus and potassium concentrations by 43% and 41%, respectively.

**Figure 3:**
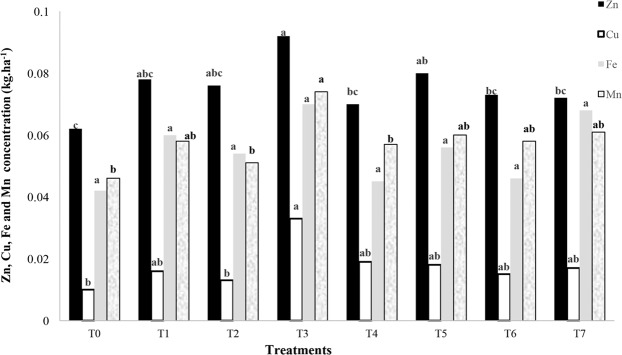
Zinc, copper, iron and manganese concentrations of white rice in response to different nano chelated iron fertilizer applications.

According to Table 4, in spite of the lack of significant difference, zinc, copper, iron and manganese concentrations of white rice in T3 were at the highest level (Fig. 3). T0 and T3 had the minimum and maximum amounts of zinc, copper, iron and manganese concentrations with 0.062; 0.092 kg/ha, 0.01; 0.033 kg/ha, 0.042; 0.07 kg/ha and 0.046; 0.074 kg/ha, respectively.

### Effect of nano chelated iron fertilizer on paddy rice macro and micronutrients content

**Figure 4:**
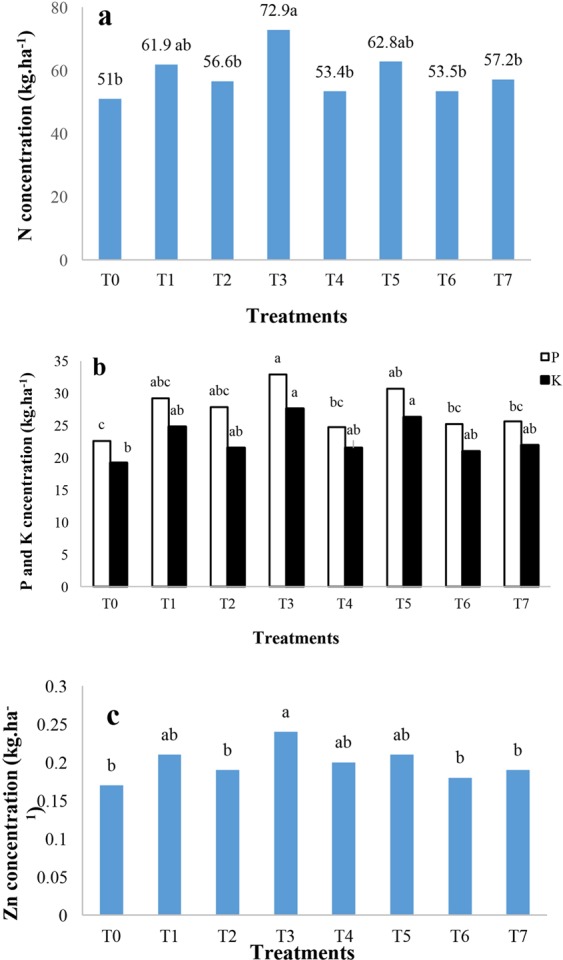
The effect of nano chelated iron fertilizer on nitrogen **(a)**, phosphorus and potassium **(b)** and zinc concentrations **(c)** of paddy rice.

**Figure 5:**
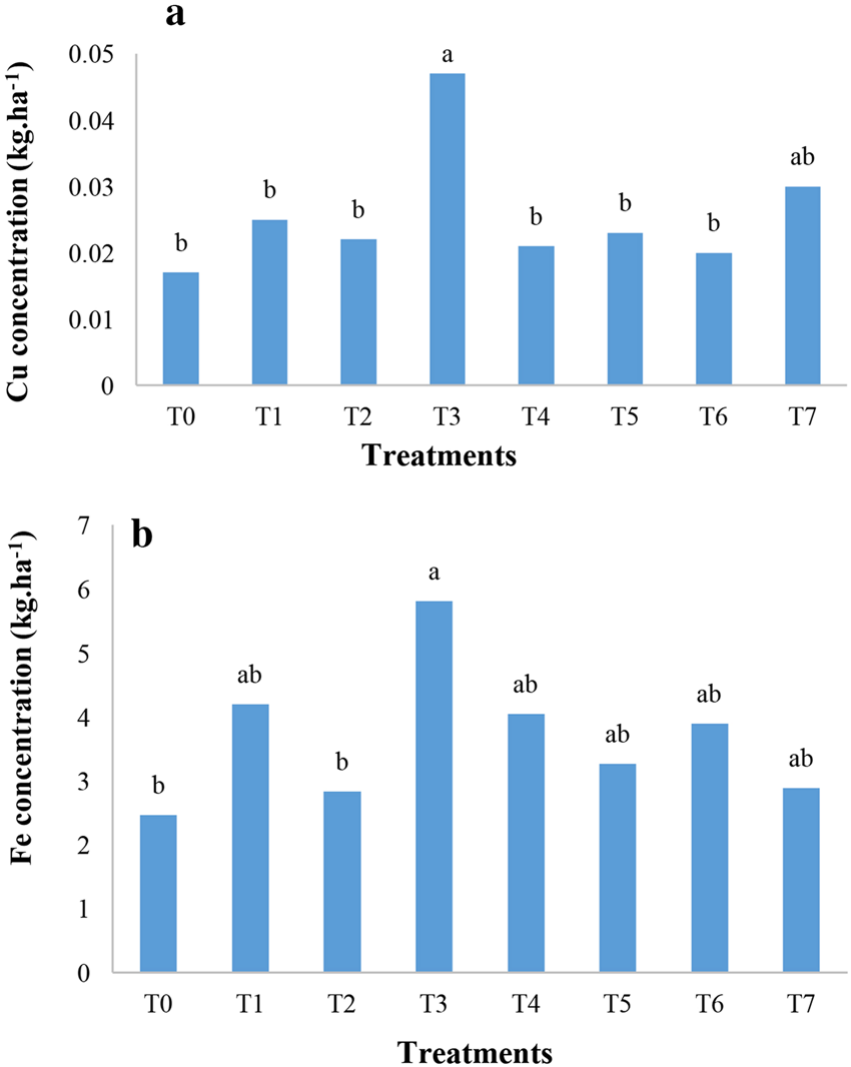
The effect of nano chelated iron fertilizer on copper **(a)** and iron concentrations **(b)** of paddy rice.

**Figure 6:**
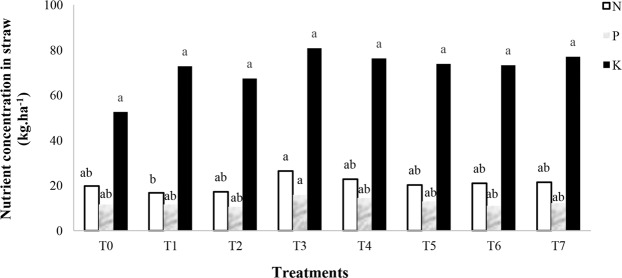
The effect of nano chelated iron fertilizer on nitrogen, phosphorus and potassium concentrations of rice straw. Nano chelated iron fertilizer was applied to evaluate nutrient changes in rice straw. Figure 6 shows that nitrogen, phosphorus and potassium concentrations did not response to nano chelated iron fertilizer.

Paddy analysis demonstrated that nitrogen concentration was impacted by nano chelated iron fertilizer application significantly (Table 5). The results of the analysis showed that T3 had the maximum amount of nitrogen content in the paddy rice with 72.9 kg/ha (Fig. 4a), and by contrast no significant effect was observed on phosphorus and potassium concentrations under other nano chelated iron fertilizer treatments (Fig. 4b). T3 showed the maximum amount of zinc, copper and iron concentrations in paddy rice (Figs. 4c, 5a,b respectively). In addition, Fig. 6 shows that nano chelated iron fertilizer application did not have any significant effect on nitrogen, phosphorus and potassium concentrations of rice straw (Table 6).

**Table 5:** Analysis of variance of N, P, K, Zn, Cu, Fe and Mn concentrations of paddy rice.

Source of changes	Freedom degree	N concentration (kg.ha^−1^)	P concentration (kg.ha^−1^)	K concentration (kg.ha^−1^)	Zn concentration (kg.ha^−1^)	Cu concentration (kg.ha^−1^)	Fe concentration (kg.ha^−1^)	Mn concentration (kg.ha^−1^)
Block	2	375.64**	50.88*	22.07 ns	0.00321*	0.00002 ns	32.46**	1.15**
Fertilizers	7	149.86*	35.64 ns	25.47 ns	0.00149 ns	0.00027 ns	3.45 ns	0.16 ns
Error	14	48.90	13.46	12.14	0.00064	0.00013	2.29	0.14
CV(%)		11.9	13.4	15.2	12.7	44	41.1	45.5

• **Significant at P ≤ 0.01, *Significant at P ≤ 0.05, ns: No significant difference.

It shows the analysis result of nutrients concentration of paddy rice. Nitrogen concentration was positively affected by nano chelated iron fertilizer.

**Table 6:** Mean square of nutrients concentration of rice straw.

Source of changes	Freedom degree	N concentration (kg.ha^−1^)	P concentration (kg.ha^−1^)	K concentration (kg.ha^−1^)
Block	2	59.20 ns	0.73 ns	14.38 ns
Fertilizers	7	28.39 ns	9.57 ns	226.58 ns
Error	14	28	7.77	285.28
CV(%)		25.7	22.1	23.5

**Significant at P ≤ 0.01, *Significant at P ≤ 0.05, ns: No significant difference.

Rice straw was tested to investigate the nutrients concentration variation. The results showed that nutrients concentration was not affected by nano chelated iron fertilizer.

## Discussion

As the previous studies have demonstrated that the application of nano chelated iron fertilizer improves both quality and quantity in various crops, it can be argued that this fertilizer, which is synthesized based on nanochelating technology, can greatly improve the delivery of this vital element to plants. In one study by Pirzad *et al*., it was revealed that using nano chelated iron fertilizer positively affects sub stem, single leaf area, and leaf weight and number of Calendula officinalis L. under water stress condition^29^.

Rezaeei *et al*. indicated that foliar application of 2.5 kg of nano chelated iron fertilizer had the highest impact on yield-related characteristics of wheat^30^. In addition, Maleki Farahani *et al*. demonstrated that application of 5 kg of nano chelated iron fertilizer led to increased yield in dry stigma, dry leaf, concentration of leaf iron and total iron of Crocus sativus L.^31^.

Applying nano chelated iron fertilizer improved all yield-related parameters in the current study. In an investigation on wheat cultivars, Harsini *et al*. (2014) itemized that plant height, spike number, 1000-grain weight, grain number per spike, biological yield and harvest index were significantly affected by nano chelated iron fertilizer as well as grain yield increase up to 20% compared to control^32^. Amuamuha *et al*. (2012) evaluated the effect of three concentrations of nano iron fertilizer (1, 2 and 3 g.l^−1^) on stem elongation, flowering and harvest time, and reported that using 1 g/L of iron nanoparticles at stem elongation stage had the highest impact on yield and essential oil. In another study on rice cultivars, Ghasemi Lemraski *et al*. (2017) well documented that paddy yield, tiller number, plant height, panicle length and harvest index were positively influenced by nano chelated iron fertilizer application.

It should be noted that rice yield is a complex trait multiplicatively determined by three component traits: number of panicles, number of grains per panicle and grain weight, all of which are typical quantitative traits. Among these characteristics, grain number is the most important determining factor in rice yield^33,34^, and also improvement in grain filling has still remained as one challenging issue^35^. However, in the present study, nano chelated iron fertilizer decreased hallow grains (by approximately 254%), while simultaneously increased other yield-related parameters^35^. Similarly, in another study by Peyvendi *et al*., it was demonstrated that using nano chelated iron fertilizer simultaneously improved growth indices and quantitative and qualitative parameters of basil (Ocimum basilicum L.).

In the present study, nano chelated iron fertilizer significantly impacted nitrogen, phosphorus and potassium concentrations of white rice in comparison to control (Table 4). Urea is the most common source of nitrogen and a hundred million tons of it is annually used worldwide, yet due to the leaching and volatilization problems, a large amount of it is wasted, which finally causes environmental pollution^36^. Based on the results of the present study, applying nano chelated iron fertilizer can improve nitrogen absorption and optimize urea fertilizer consumption. In addition to this improvement, phosphorus and potassium concentrations were positively affected by nano chelated iron fertilizer, which shows that this nano fertilizer can improve the absorption rate of phosphorus and potassium fertilizers and may prevent their abundant consumption. Likewise, one study by Vattani *et al*. showed that applying nano chelated iron fertilizer in two varieties of spinach increased the accumulation of iron and potassium, but decreased sodium, nitrate and nitro in spinach leaves^37^.

In terms of bio-fortification, multiple solutions have been suggested and used for rice bio-fortification, although most of them have shown limited results and are generally costly and unacceptable due to the changes made to rice^38^. On the other hand, genetic and agronomic bio-fortification are currently considered to be two important agricultural tools to improve rice grain nutrients concentrations. However, genetic manipulation with the aim of increasing a particular element can induce negative effects on production quantity or other nutrients content, while in the best cases it does not impact production quantity^39^. In addition, using common iron fertilizers presents its own set of problems. It is well established that increased iron uptake in rice leads to increased growth and seed yield, but not necessarily increased iron loaded in the seeds^40^.

In the present study, nano chelated iron fertilizer increased micronutrients in addition to protein content and yield parameters. According to the results of this study, protein content and zinc, copper, iron and manganese concentrations of white rice in T3 were at the highest level. This result shows that without any genetic manipulation and just by applying nano chelated iron fertilizer, rice bio-fortification would be possible. Similarly, Salarpour *et al*. reported that nano chelated iron fertilizer has positive effect on both growth characteristics and oil essence of cress at the same time^41^. Hokmabadi *et al*. also indicated that using nano chelated iron fertilizer for pistachio trees (soil and foliar application) increased the amounts of iron and calcium in fruit as well as the percentage of soluble sugar by about 40%^42^.

## Conclusion

The present study shows that foliar application of nano chelated iron fertilizer in paddy fields offers a practical and functional approach for bio-fortification of rice. Nano chelated iron fertilizer increased plant height, panicle length, grain weight and paddy yield, and in addition enriched white rice in nitrogen, phosphorus and potassium concentrations significantly as compared to control.

Increased macronutrients and protein concentration of rice by nano chelated iron fertilizer application shows that applying this fertilizer increases the uptake of macronutrients and can therefore decrease the consumption of chemical fertilizers. Foliar Application of 2.5 g/L of nano chelated iron fertilizer at nursery and booting stages had the maximum effect on rice quality and quantity parameters, all of which were obtained with a low cost and little amount of nano chelated iron fertilizer application. In conclusion, without adding more chemical fertilizers, which have hazardous effects on human health, more crops with better quality can be produced.

## Data Availability

All datasets used and/or analyzed during the current study are available on request.
